# Continued Increase in Cost of Care Despite Decrease in Stay After Posterior Spinal Fusion for Adolescent Idiopathic Scoliosis

**DOI:** 10.5435/JAAOSGlobal-D-21-00192

**Published:** 2022-03-11

**Authors:** K. Aaron Shaw, Brittany Ange, Varghese George, Joshua S. Murphy, Nicholas D. Fletcher

**Affiliations:** From the Department of Orthopaedic Surgery, Dwight D. Eisenhower Army Medical Center, Fort Gordon, GA (Dr. Shaw); the Department of Surgery, Uniformed Services University of Health Sciences, Bethesda, MD (Dr. Shaw); the Department of Biostatistics and Epidemiology, (Dr. Ange, Dr. George), Augusta University, Augusta, GA (Dr. Ange, and Dr. George); the Department of Pediatric Orthopaedic Surgery, Children's Healthcare of Atlanta–Scottish Rite, Atlanta, GA (Dr. Murphy); and the Department of Pediatric Orthopaedic Surgery, Children's Healthcare of Atlanta–Egelston, Emory University Atlanta, Atlanta, GA (Dr. Fletcher).

## Abstract

**Introduction:**

Previous studies have demonstrated decreased hospital length of stay (LOS) for children undergoing posterior spinal fusion (PSF) for adolescent idiopathic scoliosis (AIS).

**Methods:**

Hospitalization event data from the Kids Inpatient Database were queried for all PSF events for AIS performed in 2009, 2012, and 2016 using diagnosis and surgical codes. Data were subdivided into two groups: pre–enhanced recovery after surgery (ERAS) (2009 and 2012) and post-ERAS (2016). The primary outcome variables were LOS and total treatment charge (adjusted for 2020 inflation). Univariate and multivariate analysis were performed to identify differences in outcome variables.

**Results:**

A total of 12,010 unique hospitalization events were identified, 74% female, mean 14.3 years. There was a decrease in LOS (pre-ERAS: 5.4 ± 4.0 versus 4.3 ± 3.2 days, *P* < 0.0001) with an increase in adjusted total treatment charge (pre-ERAS $193,544.4 ± $108,116.1 versus $200,469.1 ± $110,112.6; *P* = 0.0013). Pre-ERAS, male sex, smaller hospital, and non-Medicaid insurance were predictive of longer LOS, whereas pre-ERAS, older age, non-White race, male sex, hospital outside the Northeast, and non-Medicaid insurance were predictive of higher treatment costs.

**Discussion:**

There continues to be a significant decrease in LOS for PSF hospitalization events for AIS; however, total treatment charges continue to rise. Future research should investigate potential factors influencing total treatment charges after PSF for AIS.

The advent of enhanced recovery after surgery (ERAS) pathways has seen significant growth across the surgical specialties. These pathways have demonstrated the ability to decrease opioid medication usage and hospital length of stay (LOS).^1,2,3,4^ This has been particularly true for children undergoing posterior spinal fusion (PSF) for adolescent idiopathic scoliosis (AIS) where multimodal pain management strategies have been incorporated with early mobilization and have resulted in significant decreases in opioid medication and hospital LOS without differences in postdischarge complications.^5,6,7,8,9,10,11,12,13^

There has been a significant increase in publications for ERAS application in patients with AIS since the initial report in 2014.^4^ The healthcare utilization and economic benefits of ERAS protocols suggest the potential for a significant societal effect^5,6,7,8,14,15^; however, no study to date has investigated the nationwide effect of ERAS pathway utilization on the US healthcare system. This study sought to investigate the effect of ERAS pathways after PSF in AIS on LOS and hospital charges in the US healthcare system. We hypothesized that the pervasiveness of ERAS pathways would result in significant decreases in LOS and hospital charges after PSF for AIS.

## Methods

This study was deemed exempt from review by the institutional review board.

### Data Source

Data were reviewed from annual hospitalization events for years 2009, 2012, and 2016 from the Kids Inpatient Database (KID). KID represents the largest pediatric accessible all-payer inpatient healthcare database in the United States. KID was created and is managed by The Agency for Healthcare Research and Quality's Healthcare Cost and Utilization Project. KID sampling includes complicated and uncomplicated births, as well as other pediatric inpatient procedures from community, nonrehabilitation hospitals in the United States. The KID database contains 107 data elements, using the *International Classification of Diseases, Ninth and 10th Revision, Clinical Modification* (*ICD-9* and *ICD-10*) formatted to code all the diagnoses and procedures. Databases are published on approximately 3-year cycles with over three million hospital stays available for each 3-year database. KID is designed to allow accurate calculation of medical condition incidences using Healthcare Cost and Utilization Project–provided trend weights. A detailed overview of the KID design is available at https://www.hcup-us.ahrq.gov/kidoverview.jsp.

### Patient Selection

Hospitalization event data from the KID database for surgical intervention for AIS were identified by *ICD* codes. Databases are published on approximately 3-year cycles, with the 2016 database representing the most current available data. Data were selected to review for year groups 2009, 2012, and 2016. For data from 2009 and 2012 data sets, *ICD-9* code 737.30 was used, and *ICD-10* code M41.12X was used for 2016 data. *ICD* procedural codes were used to include all hospitalizations corresponding to a PSF procedure (8104, 8105, 8106, 8107, 8108, 8451, 8452, and 8459). Hospitalization events were excluded from inclusion if they were <10 years of age at the time or surgery or >18 years. All hospitalization events corresponding to an anterior spinal fusion were also excluded or demonstrated a nonidiopathic scoliosis diagnosis.

The ERAS pathway was first introduced to the pediatric spinal deformity community in 2014 and consists of preferential admission to the hospital floor with early Foley and drain discontinuation, mobilization the day after surgery, combined with early institution of oral feeding, and transition to oral pain medications.^4^ As such, patient data were subdivided into two cohorts: (1) 2009 and 2012 and (2) 2016. Data including national rates, hospital costs, hospital charges, and LOS were investigated. Costs reflect the expenses incurred in the production of hospital services, whereas charges represent hospital billing. Potential confounding variables for hospital charges and LOS were collected, including age at the time of surgery, ethnicity, obesity diagnosis, and sex. Additional variables unique to patient socioeconomic status, region of treatment, hospital setting and size, and patient insurance type were also collected.

### Statistical Analysis

SAS 9.4 was used for all statistical analyses. The significance level was set at 0.05. Descriptive statistics were calculated for all qualitative and quantitative variables. The outcomes of interest were LOS and total treatment charge. Total treatment charge was adjusted for inflation rates and normalized to 2020 rates. Independent variables of interest were age, race, sex, obese (comorbidity or no comorbidity), geographic region of hospital, hospital bedsize (small, medium, and large [the definition of each bedsize category varies based on the geographic region of the hospital, in location, and teaching status]), hospital location and teaching institution (rural, urban nonteaching, or urban teaching), insurance type (Medicare, Medicaid, private insurance, self-pay, no charge, or other), median household income by zip code (0 to 25th percentile, 26th to 50th percentile, 51st to 75th percentile, or 76th to 100th percentile), and volume (number of discharges in the sample for the stratum).

Individuals from 2009 and 2012 were combined into one cohort (pre-ERAS), whereas the 2016 was defined as the post-ERAS cohort. Because of the non-normal nature of the data, Mann-Whitney *U* tests were calculated to examine differences in LOS and total treatment charge across the two cohorts. To examine the differences in the two cohorts on the outcomes while controlling for the independent variables of interest, individual multiple linear regression models were calculated. Each independent variable was first examined in a bivariate model on each of the outcomes. Independent variables that were significant at the 0.1 alpha level in a simple linear model were used to build a multivariable linear regression model. Akaike Information Criteria (AIC) were examined for each possible model. Smaller AIC values indicate a better fit, so the model with the lowest AIC was determined to be the best model for each outcome variable.

## Results

**Figure 1:**
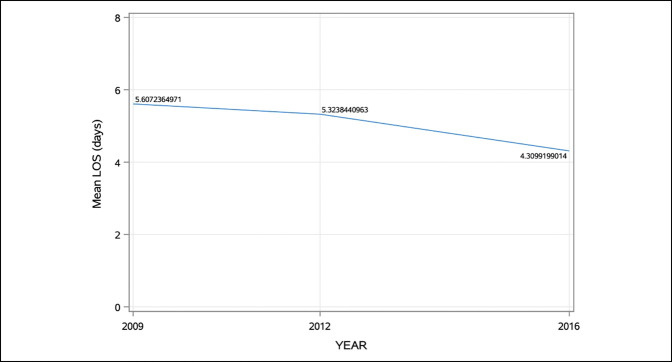
Graph showing the summary of mean hospital length of stay after posterior spinal fusion for adolescent idiopathic scoliosis according to year group.

**Figure 2:**
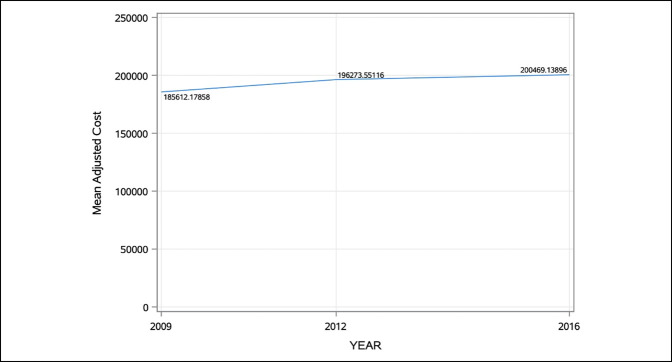
Graph showing the summary of total hospital charge, adjusted for inflation to the 2020 year rates, after posterior spinal fusion for adolescent idiopathic scoliosis according to year group.

A total of 12,010 hospitalization events for PSF were identified, corresponding to a diagnosis of AIS over the 3-year groups. Table 1 provides the descriptive statistics for the dependent variables according to the overall cohort, as well as subdivided according to the pre-ERAS and post-ERAS cohorts. The mean age in the sample was 14.4 years (SD = 2.2). The mean hospital LOS was 5.0 days (SD = 3.7), with a trend toward decreasing hospital LOS by year group, Figure 1. The mean adjusted total treatment charge was $196,392.2 (SD = $108,990.1), with a trend for increasing adjusted total treatment charge by year group, Figure 2. Most hospitalization events in the sample were Caucasian (62%) and female (76%). Overall, 59.5% of hospitalization events occurred during the pre-ERAS year groups (2009/2012), with the remained 40.5% of hospitalization events representing the post-ERAS year group (2016).

**Table 1:** Summary of Descriptive Statistics (n [%] or Mean [SD]) for Identified Variables for Hospitalization Events Associated With Posterior Spinal Fusion for Adolescent Idiopathic Scoliosis

Variable	Level	Overall, N = 12,010	Pre-ERAS, N = 7,141	Post-ERAS, N = 4,869
Year—n (%)	2009	1,907 (16%)	—	—
	2012	5,234 (44%)		
	2016	4,869 (41%)		
Length of stay—mean (SD)	—	—	5.4 (4.0)	4.3 (3.2)
Total treatment charge—mean (SD) Missing = 438	—	—	$167,846.2 (94,213.8)	$185,105.4 (101,673.6)
Adjusted total treatment charge—mean (SD) Missing = 438	—	—	$193,544.4 (108,116.1)	$200,469.1 (110,112.6)
Age—mean (SD)	—	—	14.3 (2.2)	14.4 (2.1)
Volume—mean (SD)	—	—	273,847.3 (129,903.6)	287,393.3 (140,104.5)
Race—n (%) Missing = 1,121	White	—	4,033 (61.8%)	2,752 (63.1%)
	Black	—	1,001 (15.3%)	668 (15.3%)
	Hispanic	—	792 (12.1%)	549 (12.6%)
	Asian or Pacific Islander	—	196 (3.0%)	130 (3.0%)
	Native American	—	16 (0.3%)	13 (0.3%)
	Other	—	487 (7.5%)	252 (5.8%)
Sex—n (%) Missing = 6	Male	—	1,789 (25%)	1,131 (23.2%)
	Female	—	5,348 (74.9%)	3,736 (76.8%)
Obese—n (%) Missing = 8,857 ^a^Variable is not available for 2016	Comorbidity not present	—	3,059 (97.0%)	—
	Comorbidity present	—	94 (3%)	—
Hospital region—n (%)	Northeast	—	1,116 (15.6%)	935 (19.2%)
	Midwest	—	1,706 (23.9%)	994 (20.4%)
	Southern	—	2,643 (37.0%)	1,986 (40.8%)
	Western	—	1,676 (23.5%)	954 (19.6%)
Hospital bedsize—n (%) Missing = 72	Small	—	1,142 (16.2%)	868 (17.8%)
	Medium	—	1,639 (23.2%)	973 (20.0%)
	Large	—	4,288 (60.7%)	3,028 (62.2%)
Hospital location and teaching institution—n (%) Missing = 72	Rural	—	76 (1.1%)	21 (0.4%)
	Urban nonteaching	—	514 (7.3%)	180 (3.7%)
	Urban teaching	—	6,479 (91.7%)	4,668 (95.9%)
Insurance type—n (%) Missing = 26	Medicare	—	20 (0.3%)	12 (0.3%)
	Medicaid	—	1,981 (27.8%)	1,470 (30.3%)
	Private insurance	—	4,515 (63.3%)	3,070 (63.3%)
	Self-pay	—	104 (1.5%)	59 (1.2%)
	No charge	—	88 (1.2%)	4 (0.1%)
	Other	—	425 (6.0%)	236 (4.9%)
Median household income by zip code—n (%) Missing = 219	0–25 percentile	—	1,521 (21.7%)	1,100 (23.0%)
	26–50 percentile	—	1,628 (23.2%)	1,030 (21.5%)
	51–75 percentile	—	1,781 (25.4%)	1,208 (25.3%)
	76–100 percentile	—	2,076 (29.6%)	1,447 (30.2%)

^a^The data presented only represents the data from 2009 and 2012. ERAS = enhanced recovery after surgery

A summary of univariate comparisons (Mann-Whitney *U* tests) to identify differences in hospital LOS and total adjusted treatment costs according to the pre-ERAS or post-ERAS year groups is presented in Table 2. Hospital LOS was significantly lower in the post-ERAS cohort (4.3 ± 3.2 days versus 5.4 ± 4.0 days, *P* < 0.0001). Conversely, the adjusted total treatment charge was statistically greater in the post-ERAS cohort ($185,105.4 ± 101,673.6 versus $167,846.2 ± 94,213.8 *P* = 0.0013). Tables 3 summarizes the results of linear regression results (bivariate models) on hospital LOS. Predictive variables for longer hospital LOS included pre-ERAS (slope = −1.1, *P* < 0.0001), male sex (slope = 0.4, *P* < 0.0001), non-White race (slope = 0.1, *P* = 0.0005), non-Medicaid insurance (slope = 0.1, *P* = 0.0003), and smaller hospital size (slope = −0.4, *P* < 0.0001).

**Table 2:** Summary of Univariate Mann-Whitney *U* Tests to Identify Differences in Hospital Length of Stay and Adjusted Total Treatment Charges According to Year Cohort

Variable	Pre-ERAS, N = 7,141		Post-ERAS, N = 4,869		*P*
	Mean (SD)	Median	Mean (SD)	Median	
Length of stay	5.4 (4.0)	5.0	4.3 (3.2)	4.0	<0.0001^a^
Adjusted total treatment charge ($ 2020)	$193,544.4 (108,116.1)	$172,420.6	$200,469.1 (110,112.6)	$174,039.2	0.0013^a^

ERAS = enhanced recovery after surgery

a Indicates statistical significance, *P* < 0.05.

**Table 3:** Results of Linear Regression Analysis of Identified Variables on Hospital Length of Stay

Variable	Slope	SE of Slope	*P*
Year cohort (ref = pre-ERAS)	−1.1	0.1	<0.0001^a^
Age	−0.0	0.0	0.74
Volume	0.0	0.0	0.128
Race (ref = White)	0.1	0.0	0.0005^a^
Sex (ref = F)	0.4	0.1	<0.0001^a^
Obese (ref = comorbidity present)	−0.3	0.4	0.499
Hospital region (ref = Northeast)	0.0	0.0	0.58
Hospital bedsize (ref = Small)	−0.3	0.1	<0.0001^a^
Hospital location and teaching institution (ref = urban teaching)	0.0	0.1	0.84
Insurance type (ref = Medicaid)	0.1	0.0	0.0003^a^
Median household income by zip code (ref = 0–25 percentile)	0.0	0.0	0.89

ERAS = enhanced recovery after surgery

a Indicates statistical significance, *P* < 0.05.

The results of the simple linear regression results (bivariate models) on total treatment charges, adjusted for 2020 inflation rates, are summarized in Table 4. Of the tested variables, only hospital size and presence of comorbid obesity were not statistical predictive of changes in adjusted treatment charges. The top five predictive variables for greater adjusted treatment charges include surgery in an urban teaching hospital (slope = −23688, *P* < 0.0001), surgery performed outside the Northeast United States (slope = 15602, *P* < 0.0001), male sex (slope = 13516, *P* < 0.0001), non-White race (slope = 8406, *P* < 0.0001), and post-ERAS (slope = 6294, *P* = 0.0008).

**Table 4:** Results of Linear Regression Analysis of Identified Variables on Total Treatment Charge, Adjusted for 2020 Inflation Rates

Variable	Slope	SE of Slope	*P*
Group (ref = pre-ERAS)	6294.7	2058.1	0.0008^a^
Age	1349.2	463.8	0.0036
Volume	−0.1	0.008	<0.0001
Race (ref = White)	8406.1	771.7	<0.0001^a^
Sex (ref = F)	13513.6	2357.1	<0.0001^a^
Obese (comorbidity present)	−16899.7	10890.4	0.1230
Hospital region (ref = Northeast)	15602.0	1005.8	<0.0001^a^
Hospital bedsize (ref = Small)	2540.3	1353.2	0.0605
Hospital location and teaching institution (ref = Urban teaching)	−23688.0	3497.5	<0.0001^a^
Insurance type (ref = Medicaid)	3787.6	1119.3	0.0007^a^
Median household income by zip code (ref = 0–25 percentile)	3076.2	906.6	0.0007^a^

ERAS = enhanced recovery after surgery

a Indicates statistical significance, *P* < 0.05.

The final model for LOS included ERAS year groups, race, hospital bedsize, insurance type, and sex. The final model for adjusted total treatment charge included race, sex, hospital region, hospital bedsize, hospital location and teaching, and median household income by zip code. These variables were entered into a multivariable linear regression for hospital LOS and total adjusted treatment charges (Table 5). Independent factors predictive of shorter hospital LOS included post-ERAS (*P* < 0.0001), larger hospital bedsize (*P* = 0.0028), and female sex (*P* < 0.0001). Independent factors predictive of lower total adjusted treat charge included female sex (*P* < 0.0001), hospital located in the Northeast (*P* < 0.0002), smaller hospital size (*P* = 0.0002), nonurban teaching hospital (*P* < 0.001), and Caucasian race (*P* < 0.001).

**Table 5:** Summary of Multivariable Linear Regression Models for Hospital Length of Stay and Total Adjusted Treatment Charges After Posterior Spinal Fusion for Adolescent Idiopathic Scoliosis

Outcome	Variable	Level Comparison	Slope	SE of Slope	*P*	Regression Equation
Length of stay	Intercept		5.5	0.6	<0.0001	Length of stay = 5.5 − 1.1*Group − 0.1*Race2 + 0.3*Race3 − 0.04*Race4 − 0.3*Race5 + 0.4*Race6 − 0.3*Hospital bedsize2 − 0.3*Hospitalbedsize3 + 0.1*Insurance type2 − 0.1*Insurance type3 + 1.9*Insurance type4 + 4.1*Insurance type5 + 0.1*Insurance type6 + 0.3*Sex
	Group	Post-ERAS vs. pre-ERAS^a^	−1.1	0.1	<0.0001^b^	
	Race2	Black vs. White^c^	−0.1	0.1	0.2506	
	Race3	Hispanic vs. White^d^	0.3	0.1	0.0093^b^	
	Race4	Asian or Pacific Islander vs. White^4^	−0.0	0.2	0.6683	
	Race5	Native American vs. White^e^	−0.3	0.7	0.6683	
	Race6	Other vs. White^f^	0.4	0.1	0.0069^b^	
	Hospital Bedsize2	Medium vs. small^g^	−0.3	0.1	0.0300^b^	
	Hospital Bedsize3	Large vs. small^h^	−0.3	0.1	0.0028^b^	
	Insurance type2	Medicaid vs. Medicare^i^	0.1	0.6	0.8299	
	Insurance type3	Private insurance vs. Medicare^10^	−0.1	0.6	0.8237	
	Insurance type4	Self-pay vs. Medicare^j^	1.9	0.7	0.0073	
	Insurance type5	No charge vs. Medicare^k^	4.1	0.7	<0.0001^b^	
	Insurance type6	Other vs. Medicare^l^	0.1	0.6	0.8792	
	Sex	Male vs. female^m^	0.3	0.1	<0.0001^b^	
Total adjusted treatment charge	Intercept		150076.7	6664.1	<0.0001	Cost = 150076.7 + 4619.4*Race2 + 18723.0*Race3 + 40806.3*Race4 − 56512.6*Race5 + 65779.8*Race6 + 18993.3*Sex + 6236.9*Hospital Region2 + 450.9*Hospital Region3 + 70584.6*Hospital Region4 − 6987.2*Hospital Bedsize2 + 20095.1*Hospital Bedsize3 + 59336.4*Hospital loc/teach1 + 23845.2*Hospital loc/teach2 − 2285.6*Median income2 + 5590.6*Median income3 + 9360.0*Median income4
	Race2	Black vs. White^c^	4619.4	5400.9	0.3925	
	Race3	Hispanic vs. White^d^	18723.0	6028.1	0.0019^b^	
	Race4	Asian or Pacific Islander vs. White^e^	40806.3	11436.2	0.0004^b^	
	Race5	Native American vs. White^f^	−56512.6	54293.4	0.2980	
	Race6	Other vs. White^h^	65779.8	5942.1	<0.0001^b^	
	Sex	Male vs. Female^m^	18993.3	4055.5	<0.0001^b^	
	Hospital Region2	Midwest vs. Northeast^n^	6236.9	4581.4	0.9365	
	Hospital Region3	Southern vs. Northeast^o^	450.9	5655.9	<0.0002^b^	
	Hospital Region4	Western vs. Northeast^p^	70584.6	5648.5	<0.0001^b^	
	Hospital Bedsize2	Medium vs. small^q^	−6987.2	5967.4	0.2417	
	Hospital Bedsize3	Large vs. small^r^	20095.1	5332.1	0.0002^b^	
	Hospital location and teaching1	Rural vs. urban teaching^s^	59336.4	13443.5	<0.0001^b^	
	Hospital location and teaching2	Urban nonteaching vs. urban teaching^t^	23845.2	7245.8	0.0010^b^	
	Median household income by zip code2	$25k-35k vs. $1-25k^u^	−2285.5	5496.2	0.6776	
	Median household income by zip code3	$35k-45k vs. $1-25k^v^	5590.6	5428.8	0.3032	
	Median household income by zip code4	$45k + vs. $1-25k^w^	9359.9	5234.3	0.0739	

ERAS = enhanced recovery after surgery

a Indicates statistical significance, *P* < 0.05.

b If group = 2016, enter a 1 into the equation; if group = 2009/2012, enter a 0.

c If race = Black, enter a 1 into the equation for Race2; else, enter a 0.

d If race = Hispanic, enter a 1 into the equation for Race3; else, enter a 0.

e If race = Asian or Pacific Islander, enter a 1 into the equation for Race4; else, enter a 0.

f If race = Native American, enter a 1 into the equation for Race5; else, enter a 0.

g If race = Other, enter a 1 into the equation for Race6; else, enter a 0.

h If hospital bedsize = medium, enter a 1 into the equation for hospital bedsize2; else, enter a 0.

i If hospital bedsize = large, enter a 1 into the equation for hospital bedsize3; else, enter a 0.

j If insurance type = Medicaid, enter a 1 into the equation for insurance type2; else, enter a 0.

k If insurance type = private, enter a 1 into the equation for insurance type3; else, enter a 0.

l If insurance type = self-pay, enter a 1 into the equation for insurance type4; else, enter a 0.

m If insurance type = no charge, enter a 1 into the equation for insurance type5; else, enter a 0.

n If insurance type = other, enter a 1 into the equation for insurance type6; else, enter a 0.

o If sex = male, enter a 1 into the equation for sex; else, enter a 0.

p If hospital region = Midwest, enter a 1 into the equation for hospital region2; else, enter a 0.

q If hospital region = Southern, enter a 1 into the equation for hospital region3; else, enter a 0.

r If hospital region = Western, enter a 1 into the equation for hospital region4; else, enter a 0.

s If hospital location and teaching = rural, enter a 1 into the equation for hospital location and teaching1; else, enter a 0.

t If hospital location and teaching = urban nonteaching, enter a 1 into the equation for hospital location and teaching2; else, enter a 0.

u If median household income by zip code = $25,000 to $34,999, enter a 1 into the equation for median household income by zip code2; else, enter a 0.

v If median household income by zip code = $35,000 to $44,999, enter a 1 into the equation for median household income by zip code3; else, enter a 0.

w If median household income by zip code = $45,000 or more, enter a 1 into the equation for median household income by zip code4; else, enter a 0.

## Discussion

The implementation of ERAS pathways after PSF for AIS has been shown to decrease hospital LOS and result in lower treatment charges. In this study using a nationwide database, surgeries performed before the advent of ERAS pathways demonstrated longer hospital LOS but were also associated with lower adjusted total treatment charges. Independent risk factors for shorter hospital LOS included surgery during the post-ERAS cohort, female sex, and larger hospital bedsize. Surgery performed in the Northeastern United States, nonurban teaching hospital setting, female sex, and Caucasian patient were predictive of lower adjusted treatment charge.

The length of hospital stay after PSF has significantly changed since the procedure was initially introduced for the treatment of scoliosis. No longer is LOS measured in weeks^16^ but is now measured in days.^5,6,7,8,11,14^ The introduction of ERAS protocols for AIS surgery, including multimodal pain management strategies, has been shown to produce substantial reductions in LOS but also improved pain control, faster postsurgical recovery, and lower opioid medication requirement.^4,5,17^ As the adoption of ERAS protocols has shown growing implementation since their introduction,^17^ we hypothesized that hospital LOS would significantly decrease between year cohorts and was supported with a mean 1.1 day decrease in hospital LOS in the post-ERAS cohort (4.3 ± 3.2 days versus 5.4 ± 4.0 days, *P* < 0.0001).

Additional factors were found to influence the duration of hospital LOS, including male sex, non-White race, non-Medicaid insurance, and smaller hospital size, all being associated with longer LOS. The role of sex on hospital LOS after surgery has been previously reported following various orthopaedic surgeries.^18^ Specific to PSF for AIS, few previous studies have focused on sex differences in LOS. Elsamadicy et al^19^ reported that female sex was an independent predictor of prolonged hospital LOS using the KID database. In contrast to these findings and using a substantially larger population, we found that male sex was not only predictive of longer hospital LOS but was also predictive of greater total adjusted treatment charges.

Treatment costs after PSF for AIS have been of growing concern given continued and persistent increases in mean hospital charges over time.^20,21^ Vigneswaran et al^20^ reviewed the mean treatment charges for children with AIS undergoing spinal fusion, including both anterior and posterior approaches, from 1997 until 2012 using KIDS. Treatment charges were found to increase from a mean of $55,495 in 1997 to $177,176 in 2012, resulting in sum cost of over $1.1 billion dollars in 2012. Martin et al^21^ performed a similar analysis using the NIS database, querying data from 2001 until 2011. They found that whereas the utilization rates of spinal fusion for AIS remained constant of the 10-year period assessed, treatment charges demonstrated a 113% increase from $72,780 in 2001 to $155,278 in 2011.

Although ERAS protocols have been shown to produce substantial decreases in treatment charges,^4^ up to 22%,^7^ this was not found to result in reduced adjusted treatment charges in the current study. In fact, the adjusted treatment charges significantly increased from a mean of $167,846.2 in the pre-ERAS cohort to $185,105.4, following the rising trends identified in previous studies.^20,21^ One possible explanation for this continued rise may be found in the cost and utilization of spinal implants. Kamerlink et al^22^ assessed the hospital cost and charge data of 125 consecutive spinal fusion cases, including anterior and posterior procedures, to treat AIS. The mean charge varied based on the Lenke curve classification but was reported at a mean $73,843 per case. The largest contributor to treatment cost was spinal implants, accounting for 29% of the overall cost, followed by intensive care unit/patient room costs (22%), operating room use (9.9%), and bone graft (6%).

Over time, there has been a trend toward the use of a greater number of spinal implants, especially pedicle screws, during PSF for AIS.^4,21,23^ The Minimize Implants Maximize Outcomes Study Group has investigated the influence of implant density on curve correction and clinical outcomes for children with AIS in the United States treated at 12 pediatric institutions. Higher implant densities (>1.8 screws per vertebral level fused) have been shown to produce slight but significant increases in curve corrections (69% versus 66%) for main thoracic curve patterns with similar but slightly improved clinical outcomes on patient-reported outcome measures in comparison to low implant density constructs (<1.4 screws per vertebral level fused).^24^ Given these slight improvements in deformity correction and outcomes; however, the question becomes whether these improvements are cost-effective for high-density implant constructs. High-density implant constructs for PSF result in significantly higher costs,^25^ and surgical implants have demonstrated continued increase in cost over time. Martin et al^21^ reviewed drivers of treatment charges related to PSF for AIS over a 4-year period, finding an annual increase in implant costs of 27.6% each year. The percentage of the total treatment charge dictated by implant cost increased from 28% to 53% over that 4-year period. Larson et al^26^ sought to investigate the potential mean reduction in treatment cost that could be achieved with the use of a lower-density implant construct in terms of cost and complications associated with malpositioned screws. The lower-density construct was predicted to result in a mean of 3.2 fewer screws per patient with a potential annual cost saving of between $11 and 20 million dollars, representing between 4% and 7% reduction in treatment costs.

Previous studies have identified variations in LOS and hospital charges based on geographic, hospital, and patient demographic variables after treatment of AIS.^27-29^ Menger et al^29^ reviewed the NIS database for children with AIS undergoing surgery, indicating that lower volume centers were associated with higher treatment charges but with shorter LOS after surgery. The current data found that higher volume centers demonstrated no difference in hospital LOS but were associated with higher adjusted treatment charges after PSF. However, larger hospital bedsize was predictive of both shorter hospital LOS despite being also predictive of greater total adjusted treatment costs.

In addition, the geographic location of surgery also influenced on total adjusted treatment charges with surgeries performed in the Northeastern United States having lower adjusted treatment charges than the remainder of the United States, despite no difference in hospital LOS. Daffner et al^27^ reviewed the PearlDiver Patient Record Database for children undergoing spinal fusion between 2004 and 2006. Children undergoing surgery in the Western United States were identified as having the highest hospital charges, with the lowest charges seen in the Southern United States. In addition, hospital LOS was highest in the Midwest at a mean 6.5 days and lowest in the South at 5.2 mean days. The current data suggest that across the United States, geographic differences in LOS have abated but geographic treatment charge differences do remain prevalent. This could be in part explained by the type of hospital performing the surgeries by location as teaching hospitals were identified as having higher treatment charges, which has been supported in the literature.^28^

The current study has several limitations that require recognition. As a longitudinal analysis of a blinded administrative inpatient database, causation of the identified trends is unable to be investigated or determined. There are numerous additional, inherent limitations with the use of a large database such as KIDS. There is a lack of clinical detail regarding the hospitalization events, ranging from curve magnitude, classification, treatment approach, and instrumentation density, all of which can have significant implications on both treatment cost and hospital LOS.^22,24^ In addition, the data set identifies surgical treatment using *ICD* procedural codes rather than the more commonly reported Current Procedural Terminology (CPT) codes. The current analysis is reliant on the accuracy of coding for diagnosis and treatment. Given the presence of coding inaccuracies as well as the potential for data transfer errors or exclusions in large database studies, this potential source of error cannot be overlooked.^30^ Given the utilization of a less familiar coding system for procedure codes, this may have an additional effect on coding accuracy.

Given the restraints of the current data set, we were unable to isolate and identify individual hospitals to identify them by the presence or absence of postoperative ERAS protocols. The surgeries performed included a nationwide collection of surgeons, introducing variation in treatment and surgical technique and the potential for indication bias. In addition, this investigation has been performed using data generated within 2 years of the first publication of ERAS pathways for AIS. To date, the 2016 data set is the most recent package available for analysis, which allows for only 1 year of potential post-ERAS data for analysis. Additional, more recent data would further assist the current analysis regarding the potential effect of ERAS protocols. Given the ongoing trend in decreased hospital LOS identified in previous studies,^20^ this difference in LOS may be a continued reflection of this trend rather than a true depiction of the nationwide effect of ERAS protocols.

In conclusion, using a large nationwide database of pediatric hospitalization events occurring in the United States, there continues to be a significant reduction in hospital LOS after PSF hospitalization events for AIS which persists since the introduction of ERAS protocols; however, the effect of ERAS protocols was not able to be isolated by the current data set. Interestingly, over the assessed time periods, the adjusted total treatment charges were found to increase rather than decrease as has been shown in previous hospital-based studies. Geographic and hospital setting variables do have a significant influence on both hospital LOS and total adjusted treatment charge after surgery. Future research should further investigate factors influencing this rise in adjusted total treatment charges to optimize the cost-effectiveness of PSF.
